# The genome sequence of American Willowherb, *Epilobium ciliatum* Raf. (Myrtales: Onagraceae)

**DOI:** 10.12688/wellcomeopenres.27847.1

**Published:** 2026-09-18

**Authors:** Markus Ruhsam, Peter M. Hollingsworth, Royal Botanic Garden Edinburgh Genome Acquisition Lab, Plant Genome Sizing Collective, Darwin Tree of Life Barcoding collective, Wellcome Sanger Institute Tree of Life Management, Samples and Laboratory team, Wellcome Sanger Institute Scientific Operations: Sequencing Operations, Wellcome Sanger Institute Tree of Life Core Informatics team, Tree of Life Core Informatics collective, Darwin Tree of Life Consortium

**Affiliations:** 1 Royal Botanic Garden Edinburgh, Edinburgh, Scotland, UK

**Keywords:** Epilobium ciliatum, American Willowherb, genome sequence, chromosomal, Myrtales

## Abstract

We present a genome assembly of *Epilobium ciliatum* (American Willowherb; Streptophyta; Magnoliopsida; Myrtales; Onagraceae). The assembly consists of two haplotypes with total lengths of 274.88 megabases and 262.95 megabases. Most of the haplotype 1 assembly (97.25%) was scaffolded into 18 chromosomal pseudomolecules. Haplotype 2 was assembled to scaffold level. The mitochondrial genome assembly has a length of 426.41 kilobases and the plastid genome assembly has a length of 160.91 kilobases. This assembly was generated as part of the Darwin Tree of Life project, which produces reference genomes for eukaryotic species found in Britain and Ireland.

## Species taxonomy

Eukaryota; Viridiplantae; Streptophyta; Streptophytina; Embryophyta; Tracheophyta; Euphyllophyta; Spermatophyta; Magnoliopsida; Mesangiospermae; eudicotyledons; Gunneridae; Pentapetalae; rosids; malvids; Myrtales; Onagraceae; Onagroideae; Epilobieae; *Epilobium*; *Epilobium ciliatum* Raf. (NCBI:txid33132)

## Background

*Epilobium ciliatum* is a perennial herb, native to America and parts of temperate East Asia ( POWO, 2026), which has become widely naturalised outside its native range, including much of Europe ( Strgulc Krajšek & Jogan, 2004). In the United Kingdom it was first recorded in 1891 and has since spread rapidly throughout Britain and Ireland, becoming the fastest-spreading and most widely distributed alien species in the region ( Kitchener, 2023). *E. ciliatum* occurs primarily in disturbed habitats, including gardens, walls, pavement cracks, felled woodland, waste ground and arable land ( Preston, 1988). Its broad ecological tolerance and ability to colonise recently disturbed ground have contributed to its successful establishment outside its native range ( Myerscough & Whitehead, 1967; Strgulc Krajšek & Jogan, 2004).

The ecological success of *Epilobium ciliatum* is associated with rapid growth and a flexible life history. Plants can overwinter as leafy basal rosettes and produce flowering shoots, while individuals germinating early in the growing season can reach flowering in approximately 5 to 6 weeks ( Myerscough & Whitehead, 1966, 1967; Preston, 1988). Reproduction occurs predominantly via the production of abundant seeds. Parker *et al.*. (1995) recorded 100% autonomous self-pollination in bagged flowers and generally very low insect visitation. The small, wind-dispersed seeds possess a tuft of hairs and can remain viable for several years, while flexible germination and rapid development facilitate establishment following disturbance ( Myerscough & Whitehead, 1966; Preston, 1988). Although sexual reproduction is central to colonisation, *E. ciliatum* can also persist vegetatively through overwintering basal rosettes and adventitious rooting at the base of shoots. The relative importance of these strategies varies with environmental conditions, with some populations effectively behaving as annuals and relying heavily on seed recruitment, while established plants can persist between growing seasons through vegetative structures ( Myerscough & Whitehead, 1967; Preston, 1988). The combination of rapid maturation, autonomous self-fertilisation, prolific production of wind-dispersed seeds and vegetative persistence allows *E. ciliatum* to exploit short-lived opportunities in disturbed habitats.

Recent genomic analyses using British material and genome-wide SNPs demonstrated that *Epilobium ciliatum* hybridises with native British *Epilobium* species ( Sawarda *et al.*, 2025). Despite possessing the AA chromosomal arrangement, unlike the BB arrangement found in British congeners, *E. ciliatum* showed a high propensity for hybridisation, indicating that chromosomal differences do not necessarily prevent gene flow ( Sawarda *et al.*, 2025).

The chromosome number of *E. ciliatum* is 2 *n* = 36 ( Raven & Moore, 1964; Yu, 2021).

We sequenced the genome of *Epilobium ciliatum* as part of the Darwin Tree of Life Project, which aims to generate high-quality reference genomes for all named eukaryotic species in Britain and Ireland to support research, conservation, and the sustainable use of biodiversity. The sequenced specimen was collected from Gorebridge, Scotland, UK (Figure 1).

**Figure 1.**
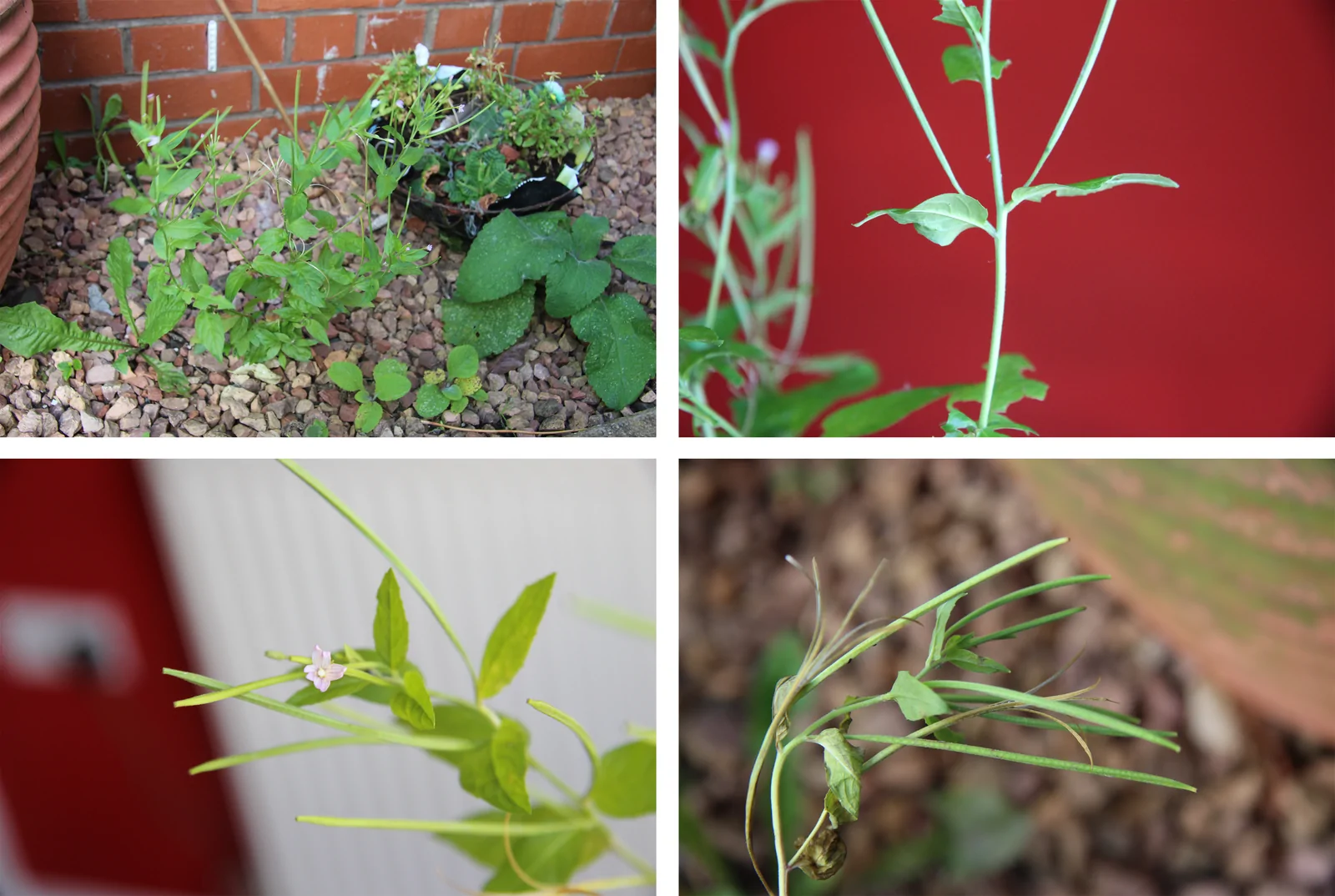
The *Epilobium ciliatum* plant sampled for genome sequencing, showing its habit (top left), stem and leaves (top right), flower (bottom left) and fruit capsules (bottom right).

## Methods

### Sample acquisition, flow cytometry and DNA barcoding

A specimen of *E. ciliatum* (specimen ID EDTOL05767, ToLID drEpiCili1; Figure 1) was used for genome sequencing. It was collected from Gorebridge, Scotland, UK (latitude 55.8431, longitude −3.0365) on 2023-08-22. The specimen was collected and identified by Markus Ruhsam. The drEpiCili1 specimen was also used for Hi-C and RNA sequencing. The herbarium voucher for the sequenced plant (collector number DMR386) is deposited in the Herbarium, Royal Botanic Garden Edinburgh (E), as specimen https://data.rbge.org.uk/herb/E01358515.

The genome size was estimated by flow cytometry following the ‘one-step’ method outlined in Pellicer *et al*. (2021) and using propidium iodide as the fluorochrome. Galbraith was used for isolation of nuclei ( Loureiro *et al.*, 2007), and the internal calibration standard was *Solanum lycopersicum* L. ‘Stupické polní rané’ with an assumed 1C-value of 968 Mb ( Doležel *et al.*, 2007).

The initial identification was verified by an additional DNA barcoding process according to the framework developed by Twyford *et al*. (2024). Part of the plant specimen was preserved in silica gel desiccant ( Chase & Hills, 1991). DNA extracted from the dried plant was amplified by PCR for standard barcode markers, with the amplicons sequenced and compared to public sequence databases including GenBank and the Barcode of Life Database (BOLD) ( Ratnasingham & Hebert, 2007). Following whole genome sequence generation, the relevant DNA barcode region was also used alongside the initial barcoding data for sample tracking at the WSI ( Twyford *et al.* , 2024). The standard operating procedures for Darwin Tree of Life barcoding are available on protocols.io.

### Nucleic acid extraction

High molecular weight (HMW) DNA extraction protocols developed by the Tree of Life Core Laboratory at the Wellcome Sanger Institute (WSI) are available on protocols.io ( Howard *et al.*, 2025). Tissue from the drEpiCili1 sample was weighed and triaged to select the appropriate extraction workflow. HMW DNA was prepared for one PacBio HiFi library: DTOL15020090, for which 87.0 mg of leaf tissue was homogenised by cryogenic bead beating. HMW DNA was extracted using the Automatic Plant Organic Extraction protocol. Sheared DNA was purified by automated SPRI (solid-phase reversible immobilisation), using AMPure PB beads (Pacific Biosciences) and the Thermo Fisher KingFisher™ Apex to eliminate shorter fragments and concentrate the DNA.

The concentration of the sheared and purified DNA was assessed with a NanoDrop spectrophotometer and a Qubit Fluorometer using the Qubit dsDNA High Sensitivity Assay kit. Fragment size distribution was evaluated by running the sample on the Femto Pulse system. For this sample, final post-shearing DNA measurements included a Qubit concentration of 14.7 ng/μL, and an average fragment size of 18.5 kb. The 260/280 spectrophotometric ratio was 1.73, and the 260/230 ratio was 1.1.

RNA was extracted from leaf tissue of drEpiCili1 in the Tree of Life Laboratory at the WSI using the RNA Extraction: Automated MagMax™ *mir*Vana protocol. The RNA concentration was assessed with a NanoDrop spectrophotometer and a Qubit Fluorometer using the Qubit RNA Broad-Range Assay kit. RNA integrity was assessed using the Agilent RNA 6000 Pico Kit and Eukaryotic Total RNA assay.

### PacBio HiFi library preparation and sequencing

Library preparation and sequencing took place in the WSI Scientific Operations core. Library DTOL15020090 was prepared with the SMRTbell Prep Kit 3.0 (Pacific Biosciences) according to the manufacturer’s instructions. The kit includes reagents for end repair/A-tailing, adapter ligation, post-ligation SMRTbell bead clean-up, and nuclease treatment. Size selection and clean-up were performed using diluted AMPure PB beads (Pacific Biosciences). DNA concentration was quantified with a Qubit Fluorometer v4.0 (Thermo Fisher Scientific) and the Qubit 1X dsDNA HS assay kit. Final library fragment size was assessed with the Agilent Femto Pulse Automated Pulsed Field CE Instrument (Agilent Technologies) using the gDNA 55 kb BAC analysis kit.

The library was sequenced on a Revio instrument (Pacific Biosciences, California, USA). The prepared libraries selected for multiplexing were pooled based on genome size, library molarity, and the intended plex level. Primers were annealed and polymerases were bound to generate circularised complexes, following the manufacturer’s instructions. Complexes were purified using SMRTbell beads, diluted to the Revio loading concentration, and spiked with a Revio sequencing internal control. The pooled libraries were sequenced on a Revio 25M Plex SMRT cell in a 4-plex run. SMRT Link software (Pacific Biosciences), a web-based workflow manager, was used to configure and monitor the run and to carry out primary and secondary data analysis.

### Hi-C

***Sample preparation and crosslinking***

Hi-C data were generated from the drEpiCili1 leaf sample using the Arima-HiC v2 kit (Arima Genomics). Tissue was finely ground using the Covaris cryoPREP Dry Pulverizer (Covaris), and then subjected to nuclei isolation. Nuclei were isolated using a modified protocol based on the Qiagen QProteome Cell Compartment Kit (Qiagen), in which only the Lysis and CE2 buffers were used, with QIAshredder spin columns. After isolation, nuclei were fixed using formaldehyde to a final concentration of 2% to crosslink the DNA. The crosslinked DNA was then digested and biotinylated according to the manufacturer’s instructions. A clean-up step was performed with SPRIselect beads before library preparation. DNA concentration was quantified using the Qubit Fluorometer v4.0 (Thermo Fisher Scientific) and the Qubit HS Assay Kit, following the manufacturer’s instructions.

***Hi-C library preparation and sequencing***

Biotinylated DNA constructs were fragmented using a Covaris E220 sonicator and size-selected to 400–600 bp using SPRISelect beads. DNA was enriched with Arima-HiC v2 kit Enrichment beads. End repair, A-tailing, and adapter ligation were carried out with the NEBNext Ultra II DNA Library Prep Kit (New England Biolabs), following a modified protocol where library preparation occurs while DNA remains bound to the Enrichment beads. Libraries were amplified with KAPA HiFi HotStart mix and a custom Unique Dual Index (UDI) barcode set (Integrated DNA Technologies). Depending on sample concentration and biotinylation percentage determined at the crosslinking stage, libraries were amplified with 10–16 PCR cycles. Post-PCR clean-up was performed with SPRISelect beads. Libraries were quantified using the AccuClear Ultra High Sensitivity dsDNA Standards Assay Kit (Biotium) and a FLUOstar Omega plate reader (BMG Labtech).

Prior to sequencing, libraries were normalised to 10 ng/μL. Normalised libraries were quantified again to create equimolar and/or weighted 2.8 nM pools. Pool concentrations were checked using the Agilent 4200 TapeStation (Agilent) with High Sensitivity D500 reagents before sequencing. Libraries were sequenced on the Illumina NovaSeq X, generating paired-end 150-bp reads.

### RNA library preparation and sequencing

Libraries were prepared with the NEBNext® Ultra II Directional RNA Library Prep Kit for Illumina (New England Biolabs), following the manufacturer’s instructions. Poly(A) mRNA was isolated from 100 ng of total RNA using oligo(dT) beads, converted to cDNA, and uniquely indexed; 14 PCR cycles were performed. Libraries were prepared with an intended average fragment size of 100–300 bp. Libraries were quantified, normalised, and pooled equimolarly to a final concentration of 2.8 nM before dilution to 150 pM for loading. Sequencing was carried out on the Illumina NovaSeq X, generating paired-end reads.

### Genome assembly

Prior to assembly of the PacBio HiFi reads, a database of *k*-mer counts ( *k* = 31) was generated from the filtered reads using FastK. The *k*-mer frequency distribution was analysed with GenomeScope (version 2.1.0) ( Ranallo-Benavidez *et al.*, 2020), providing fitted point estimates of genome size, heterozygosity and repeat content.

The HiFi reads were assembled using Hifiasm in Hi-C phasing mode ( Cheng *et al.*, 2021, 2022), producing two haplotypes. Hi-C reads ( Rao *et al.* , 2014) were mapped to contigs using bwa-mem2 ( Vasimuddin *et al.*, 2019). Contigs were further scaffolded with Hi-C data in YaHS ( Zhou *et al.*, 2023), using the --break option for handling potential misassemblies. The scaffolded assemblies were evaluated using Gfastats ( Formenti *et al.* , 2022), BUSCO ( Manni *et al.*, 2021) and MerquryFK ( Rhie *et al.*, 2020). The organelle genomes were assembled using OATK ( Zhou *et al.*, 2025). The comparator for the mitochondrial assembly was the mitochondrial genome of *Eucalyptus grandis* (NC_040010.1).

### Assembly curation

The assembly was decontaminated using the Assembly Screen for Cobionts and Contaminants ( ASCC) pipeline. The flat files and maps for curation were generated with TreeVal. Manual curation was conducted primarily in PretextView and HiGlass ( Kerpedjiev *et al.* , 2018). Scaffolds were visually inspected and corrected as described by Howe *et al*. (2021). The two haplotype assemblies were combined for curation. Manual corrections included 35 breaks and 47 joins. This reduced the scaffold count by 3.5%, increased the scaffold N50 by 12.2%, and increased the total assembly length by 0.8%. The curation process is described at sanger-tol/curation-resources. A Hi-C contact map of the haplotype 1 assembly was generated with PretextSnapshot.

### Assembly quality assessment

The MerquryFK tool ( Rhie *et al.*, 2020) was run in a Singularity container ( Kurtzer *et al.*, 2017) to evaluate *k*-mer completeness and assembly quality for both haplotypes using the *k*-mer database ( *k* = 31) computed prior to genome assembly. The analysis outputs included assembly QV scores and completeness statistics.

The haplotype 1 assembly was assessed using BlobToolKit ( Challis *et al.*, 2020). PacBio reads were mapped to the haplotype 1 assembly to derive sequence coverage, and BUSCO ( Manni *et al.*, 2021) was used to assess gene-set completeness. DIAMOND BLASTp searches of extracted BUSCO proteins and DIAMOND BLASTx searches of assembly sequences against UniProt Reference Proteomes ( Buchfink *et al.*, 2021), followed by BLASTn searches of sequences without DIAMOND BLASTx hits against the NCBI nt database ( Altschul *et al.*, 1990), supported taxonomic assignment. Coverage, sequence composition, taxonomic assignment and BUSCO results were integrated into a BlobDir for interactive inspection and visualisation. The snail and blob plots were rendered locally from this hosted BlobDir using BlobTk v0.8.3. The scaffold-length radial scale function for the snail plot was set to linear ( Challis & Blaxter, 2026). The blob plot used circular markers with area proportional to scaffold length ( Challis *et al.*, 2020).

## Genome sequence report

### Sequence data

The genome of a specimen of *E. ciliatum* was sequenced using Pacific Biosciences single-molecule HiFi long reads, generating 11.71 Gb (gigabases) from 1.09 million reads. We assembled the genome from these reads. GenomeScope analysis using a *p* = 2 model gave a *k*-mer-based haploid genome size estimate of 321.11 Mb, with heterozygosity of 0.09% and repeat content of 51.52%; the full-model fit was 97.41% (Figure 2). Using flow cytometry, the genome size (1C‐value) of the sample was estimated to be 0.36 pg, equivalent to 360.00 Mb. These estimates guided expectations for the assembly. The sequencing data provided approximately 35× coverage of the haplotype 1 assembly. Hi-C sequencing produced 152.06 Gb from 503.50 million read pairs, which were used to scaffold the haplotype 1 assembly. RNA sequencing data were also generated and are available in public sequence repositories. Table 1 summarises the specimen and sequencing details.

**Figure 2.**
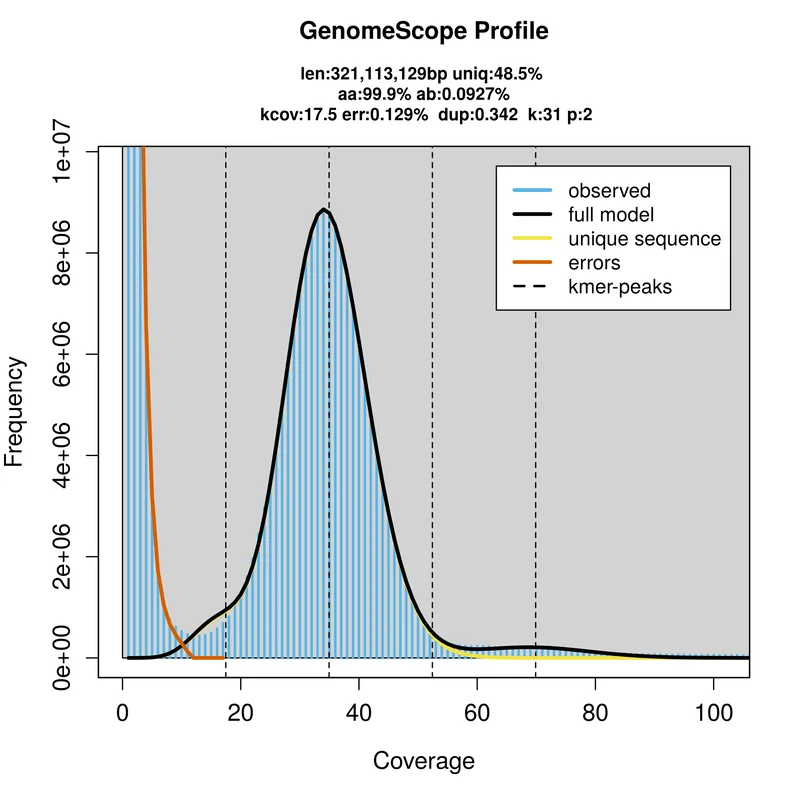
GenomeScope model fitted to the *k*-mer frequency distribution. The plot shows the observed and modelled *k*-mer spectra used to estimate genome size, heterozygosity and repeat content from unassembled sequencing reads.

**Table 1.** Specimen and sequencing data for *E. ciliatum* (BioProject PRJEB81636)

**Platform**	**PacBio HiFi**	**Hi-C**	**RNA-seq**
**ToLID**	drEpiCili1	drEpiCili1	drEpiCili1
**Specimen ID**	EDTOL05767	EDTOL05767	EDTOL05767
**BioSample (source individual)**	SAMEA114782695	SAMEA114782695	SAMEA114782695
**BioSample (tissue)**	SAMEA114788072	SAMEA114788072	SAMEA114788072
**Tissue**	leaf	leaf	leaf
**Instrument**	Revio (1 run)	Illumina NovaSeq X	Illumina NovaSeq X
**Library ID & run accession(s)**	DTOL15020090: ERR13900460	ERR13907240	ERR14379151
**Read count total**	1.09 million reads	503.50 million read pairs	51.41 million read pairs
**Base count total**	11.71 Gb	152.06 Gb	15.53 Gb

### Assembly statistics

The genome was assembled into two haplotypes using Hi-C phasing. The haplotype 1 assembly was curated to chromosome level, while haplotype 2 was assembled to scaffold level. The haplotype 1 assembly, which is proposed as the reference assembly for this species, has a total length of 274.88 Mb in 163 scaffolds (excluding organelle sequences), with 109 gaps, and a scaffold N50 of 15.14 Mb (Table 2). The haplotype 1 assembly has a scaffold auN of 14.7 Mb.

**Table 2.** Genome assembly statistics for *E. ciliatum*

**Genome assembly**	**Haplotype 1**	**Haplotype 2**
**Assembly name**	drEpiCili1.hap1.1	drEpiCili1.hap2.1
**Assembly accession**	GCA_965637465.1	GCA_965637425.1
**Assembly level**	chromosome	scaffold
**Span (Mb)**	274.88	262.95
**Number of chromosomes**	18	-
**Number of contigs**	272	199
**Contig N50 (Mb)**	3.78	3.11
**Number of scaffolds (excluding organelle sequences)**	163	92
**Scaffold N50 (Mb)**	15.14	14.58
**Scaffold auN (Mb)**	14.7	14.3
**Longest scaffold length (Mb)**	18.26	17.84
**Organelles**	Mitochondrial genome: 426.41 kb; Plastid genome: 160.91 kb	-

Most of the haplotype 1 assembly sequence (97.25%) was assigned to 18 chromosome-level scaffolds. These chromosome-level scaffolds, confirmed by Hi-C data, are named according to size (Figure 3; Table 3).

The mitochondrial genome (length 426.41 kb, OZ281309.1) and plastid genome (length 160.91 kb, OZ281310.1) were also assembled. These sequences are included as contigs in the multifasta file of the haplotype 1 genome submission and as standalone records.

**Figure 3.**
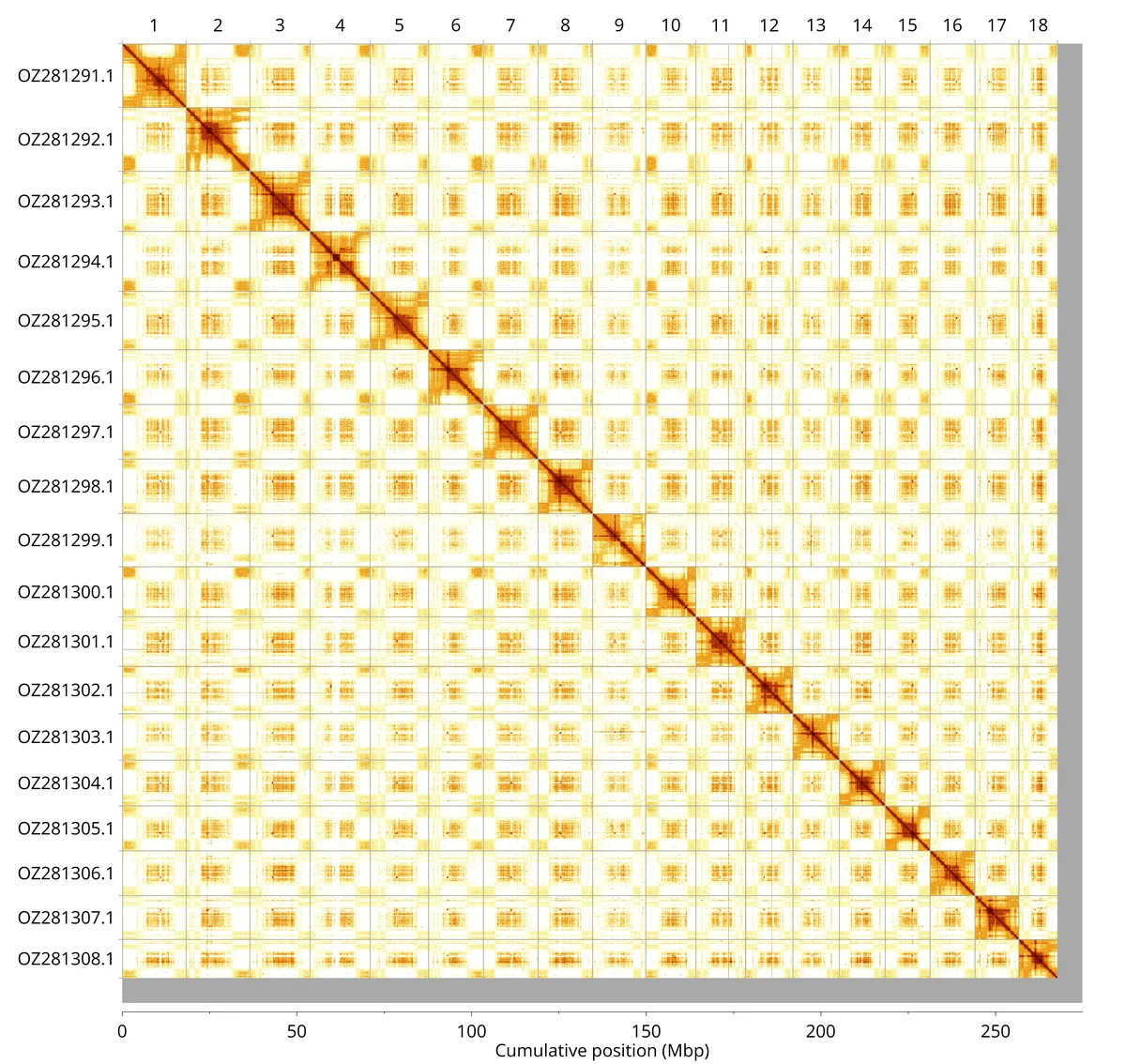
Hi-C contact map of the *E. ciliatum* haplotype 1 assembly. Assembled chromosomes are shown in order of size and labelled along the axes, with a megabase scale shown below. The plot was generated using PretextSnapshot.

**Table 3.** Chromosomal pseudomolecules in the haplotype 1 genome assembly of *E. ciliatum* drEpiCili1

**INSDC accession**	**Molecule**	**Length (Mb)**	**GC%**
OZ281291.1	1	18.26	40
OZ281292.1	2	18.22	40.50
OZ281293.1	3	17.20	41.50
OZ281294.1	4	17.15	40.50
OZ281295.1	5	16.67	41
OZ281296.1	6	15.67	40
OZ281297.1	7	15.63	41.50
OZ281298.1	8	15.61	41
OZ281299.1	9	15.14	40
OZ281300.1	10	14.34	41
OZ281301.1	11	14.16	40
OZ281302.1	12	13.57	40
OZ281303.1	13	13.27	39.50
OZ281304.1	14	13.05	40.50
OZ281305.1	15	12.84	40.50
OZ281306.1	16	12.82	40
OZ281307.1	17	12.54	39.50
OZ281308.1	18	11.15	40

### Assembly quality metrics

For the haplotype 1 assembly, the estimated QV is 65.4, and for the haplotype 2 assembly, 66.1. When the two haplotypes are combined, the assembly achieves an estimated QV of 65.7. The *k*-mer completeness is 97.50% for the haplotype 1 assembly, 96.77% for the haplotype 2 assembly, and 97.80% for the combined haplotypes (Figure 4).

BUSCO analysis using the eudicots_odb10 reference set ( *n* = 2,326) identified 96.5% of the expected gene set (single = 84.2%, duplicated = 12.3%) in the haplotype 1 assembly. For the haplotype 2 assembly, BUSCO v.6.0.0 analysis identified 96.2% of the expected gene set (single = 83.8%, duplicated = 12.3%).

**Figure 4.**
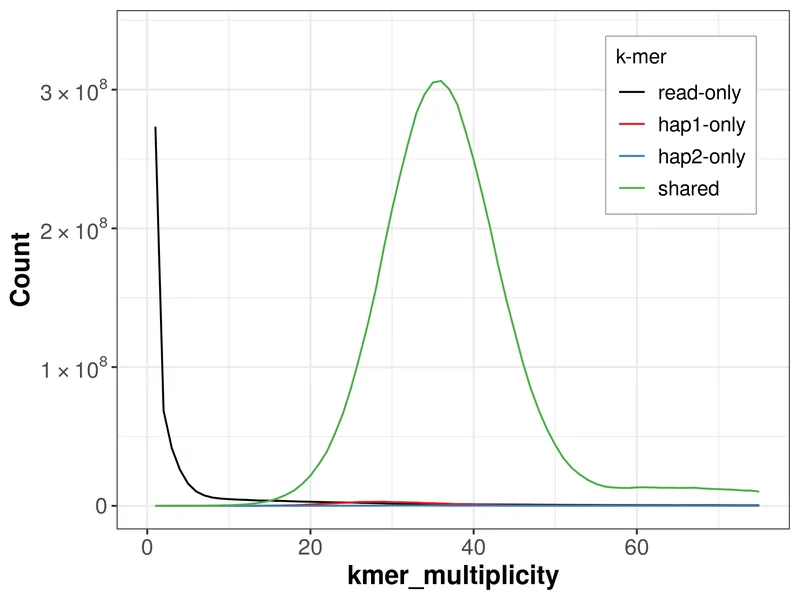
Evaluation of *k*-mer completeness using MerquryFK. This plot illustrates the recovery of *k*‐mers from the original read data in the haplotype 1 assembly, the haplotype 2 assembly and the combined haplotypes. The horizontal axis represents *k*‐mer multiplicity, and the vertical axis shows the number of *k*‐mers. The black curve represents *k*‐mers that appear in the reads but are not assembled, while the remaining curves correspond to *k*‐mers recovered in the assemblies shown.

The snail plot in Figure 5 provides a summary of assembly contiguity, cumulative scaffold count, sequence composition and BUSCO completeness for the haplotype 1 assembly ( Challis & Blaxter, 2026). The relative sizes of the longest scaffold, scaffold N50 and N90, together with the dark-grey scaffold-length distribution, show how much of the assembly is contained in long scaffolds. The outer composition track shows variation in GC, AT and N content, while the central track shows the cumulative scaffold count and the BUSCO inset summarises gene-set completeness.

The blob plot in Figure 6 displays scaffolds from the haplotype 1 assembly by GC proportion and base coverage. Marker area is proportional to scaffold length, and colour indicates taxonomic assignment ( Challis *et al.*, 2020).

**Figure 5.**
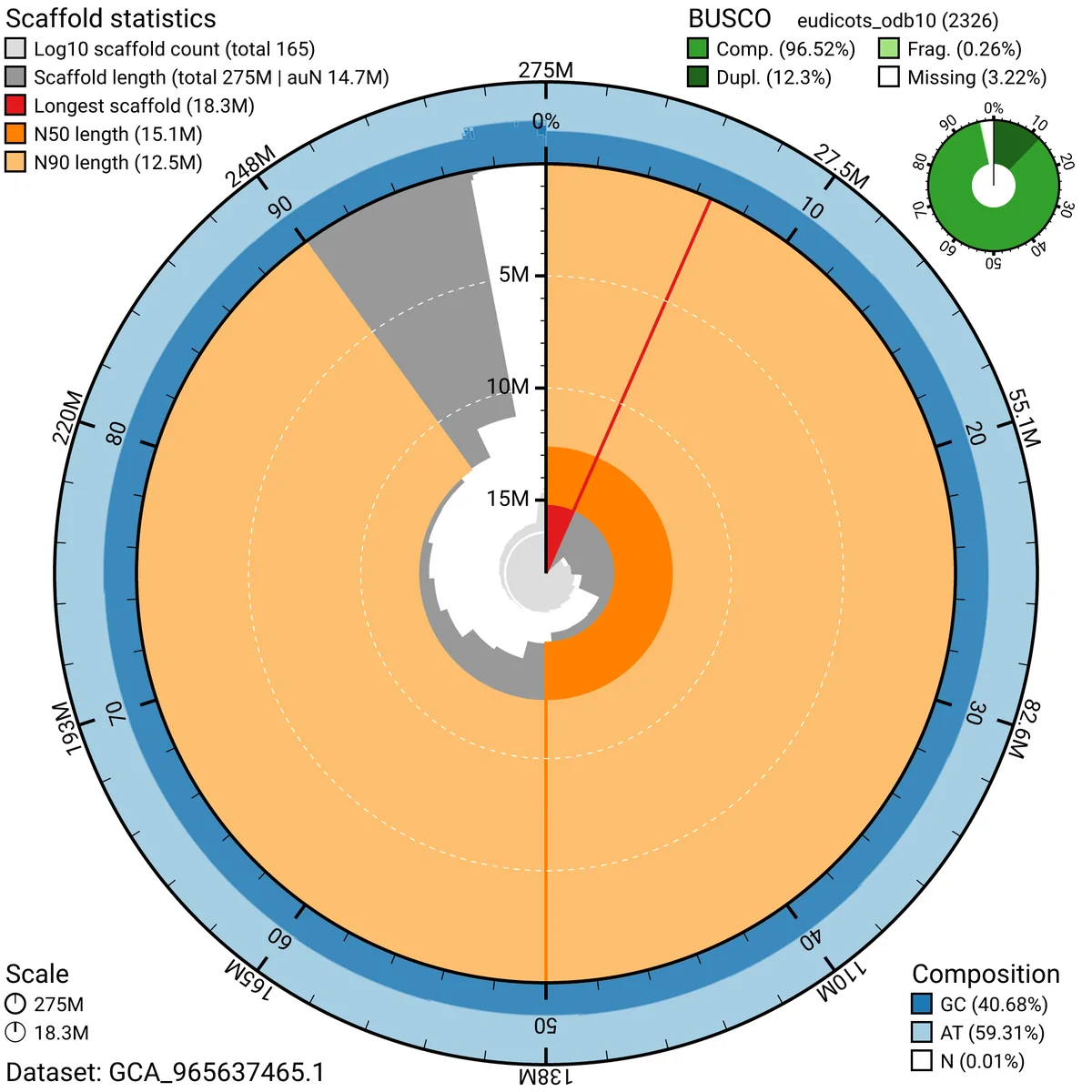
Assembly metrics for drEpiCili1.hap1.1, the haplotype 1 assembly. The BlobToolKit snail plot provides an overview of assembly metrics and BUSCO gene completeness. The circumference represents the length of the haplotype 1 assembly, and the main plot is divided into 1,000 bins around the circumference. The outermost blue tracks display the distribution of GC, AT, and N percentages across the bins. Scaffolds are arranged clockwise from longest to shortest and are depicted in dark grey. Scaffold length is shown on a linear radial scale, scaled to the longest scaffold. Red shading indicates the longest scaffold, and the deeper orange and pale orange shading represent the N50 and N90 lengths. The summary statistics include scaffold auN, the area under the scaffold Nx curve. The light grey spiral at the centre shows the cumulative scaffold count on a logarithmic scale. A summary of complete, fragmented, duplicated, and missing BUSCO genes in the eudicots_odb10 set is presented at the top right. The assembly can be explored interactively in the BlobToolKit viewer.

**Figure 6.**
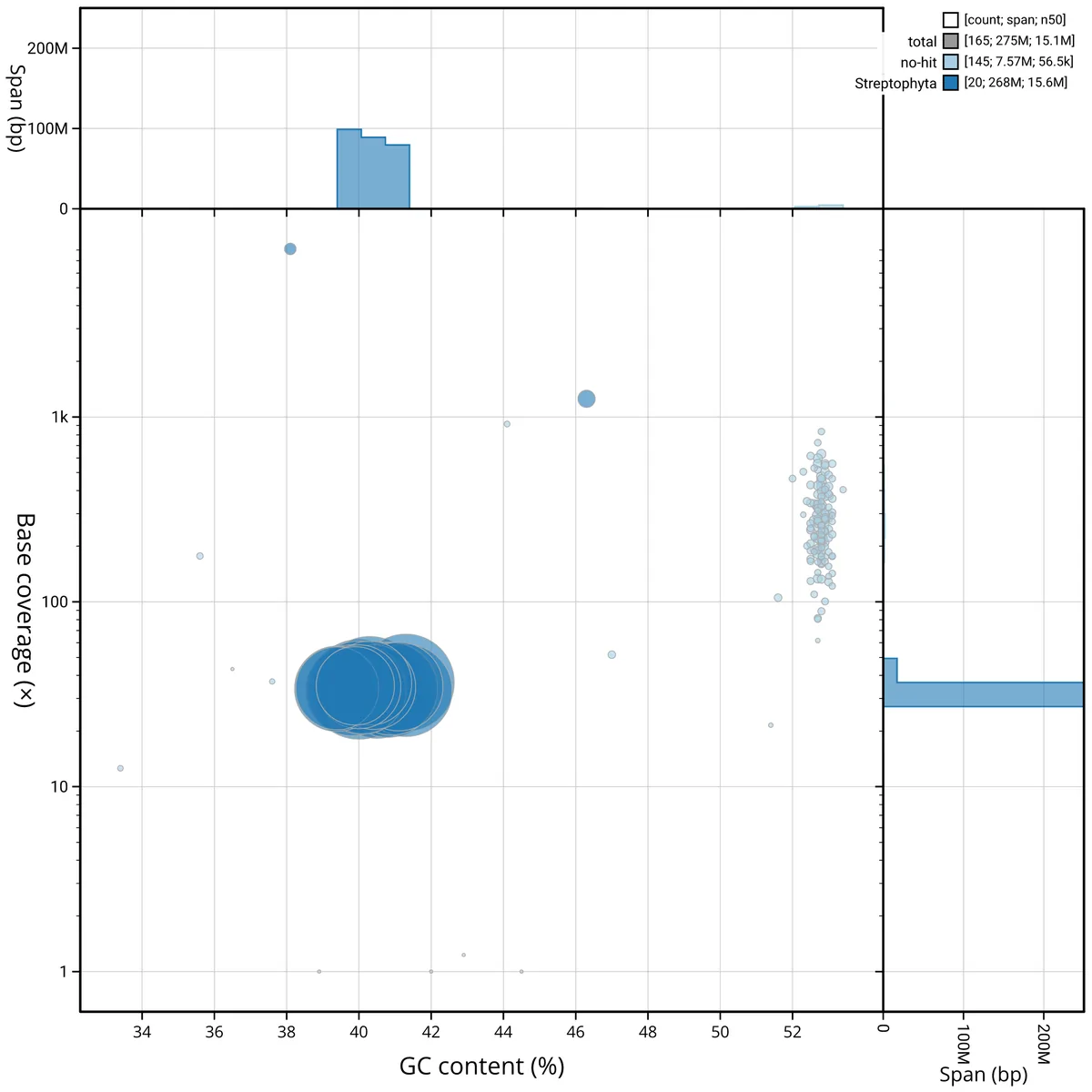
BlobToolKit blob plot for drEpiCili1.hap1.1, the haplotype 1 assembly. The plot shows base coverage (vertical axis) and GC content (horizontal axis). Circles represent scaffolds; marker area is proportional to scaffold length, and colour indicates taxonomic assignment. The histograms along the axes display the total length of sequences distributed across different levels of coverage and GC content. The assembly can be explored interactively in the BlobToolKit viewer.

Table 4 lists the assembly metric benchmarks adapted from Rhie *et al.*. (2021) and the Earth BioGenome Project (EBP) Report on Assembly Standards. The three-part EBP metric calculated for the haplotype 1 assembly is **6.C.Q65**.

**Table 4.** Earth BioGenome Project summary metrics for the *E. ciliatum* assembly

**Measure**	**Value**	***Benchmark***
EBP summary (haplotype 1)	6.C.Q65	*6.C.Q40*
Contig N50 length	3.78 Mb	*> 1 Mb*
Scaffold N50 length	15.14 Mb	*= chromosome N50*
Consensus quality (QV)	Haplotype 1: 65.4; haplotype 2: 66.1; combined: 65.7	*≥ 40*
*k*-mer completeness	Haplotype 1: 97.50%; Haplotype 2: 96.77%; combined: 97.80%	*> 90%*
BUSCO	Haplotype 1: C:96.5% [S:84.2%, D:12.3%], F:0.3%, M:3.2%, n:2,326; Haplotype 2: C:96.2% [S:83.8%, D:12.3%], F:0.3%, M:3.5%, n:2,326	*S > 90%; D < 5%*
Percentage of assembly assigned to chromosomes	97.25%	*≥ 90%*

**Notes:** The EBP summary uses log10(Contig N50); chromosome-level (C) or log10(Scaffold N50); Q (Merqury QV). BUSCO v6.0.0 using the eudicots_odb10 lineage dataset: C=complete; S=single-copy; D=duplicated; F=fragmented; M=missing; n=orthologues.

### Data availability

European Nucleotide Archive: Epilobium ciliatum. Accession number PRJEB81636; https://identifiers.org/ena.embl/PRJEB81636. The genome sequence is released openly for reuse. The *E. ciliatum* genome sequencing initiative is part of the Darwin Tree of Life Project ( PRJEB40665) and Sanger Institute Tree of Life Programme ( PRJEB43745). All raw sequence data and the haplotype assemblies have been deposited in INSDC databases. The genome will be annotated using available RNA-Seq data and presented through the Ensembl pipeline at the European Bioinformatics Institute. Raw data and assembly accession identifiers are reported in Table 1 and Table 2.

Pipelines used for genome assembly at the WSI Tree of Life are available at https://pipelines.tol.sanger.ac.uk/pipelines. Table 5 lists software versions used in this study.

**Table 5.** Software versions and sources used for *E. ciliatum*

**Software**	**Version**	**Source**
BLAST	2.14.0	https://ftp.ncbi.nlm.nih.gov/blast/executables/blast+/
BlobToolKit	4.4.6	https://github.com/genomehubs/blobtoolkit
BlobTk (hosted BlobDir)	0.5.1	https://github.com/genomehubs/blobtk
BlobTk (snail and blob plot renderer)	0.8.3	https://github.com/genomehubs/blobtk
BUSCO	6.0.0; 6.1.0	https://gitlab.com/ezlab/busco
bwa-mem2	2.2.1	https://github.com/bwa-mem2/bwa-mem2
DIAMOND	2.1.8	https://github.com/bbuchfink/diamond
fasta_windows	0.2.4	https://github.com/tolkit/fasta_windows
FastK	1.2	https://github.com/thegenemyers/FASTK
GenomeScope	2.1.0	https://github.com/tbenavi1/genomescope2.0
Gfastats	1.3.6	https://github.com/vgl-hub/gfastats
Hifiasm	0.19.8-r603	https://github.com/chhylp123/hifiasm
HiGlass	1.13.4	https://github.com/higlass/higlass
MerquryFK	1.1.0-c1	https://github.com/thegenemyers/MERQURY.FK
Minimap2	2.28-r1209	https://github.com/lh3/minimap2
Oatk	0.9	https://github.com/c-zhou/oatk
MultiQC	1.14; 1.17 and 1.18	https://github.com/MultiQC/MultiQC
Nextflow	24.10.4	https://github.com/nextflow-io/nextflow
PretextSnapshot	0.0.6	https://github.com/sanger-tol/PretextSnapshot
PretextView	1.0.3	https://github.com/sanger-tol/PretextView
samtools	1.21	https://github.com/samtools/samtools
sanger-tol/ascc	0.1.0	https://github.com/sanger-tol/ascc
sanger-tol/blobtoolkit	v0.9.0	https://github.com/sanger-tol/blobtoolkit
sanger-tol/curationpretext	1.4.2	https://github.com/sanger-tol/curationpretext
Seqtk	1.4-r122	https://github.com/lh3/seqtk
Singularity	3.9.0	https://github.com/sylabs/singularity
TreeVal	1.1.1	https://github.com/sanger-tol/treeval
YaHS	1.2a.2	https://github.com/c-zhou/yahs

### Author information

- Members of the Royal Botanic Garden Edinburgh Genome Acquisition Lab
- Members of the Plant Genome Sizing Collective
- Members of the Darwin Tree of Life Barcoding collective
- Members of the Wellcome Sanger Institute Tree of Life Management, Samples and Laboratory team
- Members of Wellcome Sanger Institute Scientific Operations – Sequencing Operations
- Members of the Wellcome Sanger Institute Tree of Life Core Informatics team
- Members of the Tree of Life Core Informatics collective
- Members of the Darwin Tree of Life Consortium

### Wellcome Sanger Institute – Legal and Governance

The materials that have contributed to this genome note have been supplied by a Darwin Tree of Life Partner. The submission of materials by a Darwin Tree of Life Partner is subject to the **‘Darwin Tree of Life Project Sampling Code of Practice’**, which can be found in full on the Darwin Tree of Life website. By agreeing with and signing up to the Sampling Code of Practice, the Darwin Tree of Life Partner agrees they will meet the legal and ethical requirements and standards set out within this document in respect of all samples acquired for, and supplied to, the Darwin Tree of Life Project. Further, the Wellcome Sanger Institute employs a process whereby due diligence is carried out proportionate to the nature of the materials themselves, and the circumstances under which they have been/are to be collected and provided for use. The purpose of this is to address and mitigate any potential legal and/or ethical implications of receipt and use of the materials as part of the research project, and to ensure that in doing so we align with best practice wherever possible. The overarching areas of consideration are:

- Ethical review of provenance and sourcing of the material
- Legality of collection, transfer and use (national and international)

Each transfer of samples is further undertaken according to a Research Collaboration Agreement or Material Transfer Agreement entered into by the Darwin Tree of Life Partner, Genome Research Limited (operating as the Wellcome Sanger Institute), and in some circumstances, other Darwin Tree of Life collaborators.
