# Supplementary material for: The genome sequence of American Willowherb, Epilobium ciliatum Raf. (Myrtales: Onagraceae)
Source: Wellcome Open Res. 2026 Sep 18;11:699. [Version 1] doi: 10.12688/wellcomeopenres.27847.1 (PMC13743839; doi:10.12688/wellcomeopenres.27847.1)
Supplement: Peer review history

# Reviewer report: Reviewer response for version 1

**DOI:** 10.21956/wellcomeopenres.30644.r175230

**Published:** 2026-11-06

**Authors:** Diksha Gambhir

**Affiliations:** 1 Boston University, Boston, Massachusetts, USA

The manuscript presents a genome assembly of willowherb, generated using standard HiFi and Hi-C assembly protocols. Haplotype 1 was assembled to the chromosome level, whereas haplotype 2 remains at the scaffold level. The current assembly lacks gene annotation, although the authors plan to generate annotations using RNA-seq data in the future. Ideally, gene annotation should be included alongside the genome assembly to provide a more comprehensive genomic resource. Nevertheless, the manuscript is overall scientifically sound and clearly written.

Are the datasets clearly presented in a usable and accessible format, and the assembly and annotation available in an appropriate subject-specific repository?

Yes

Are sufficient details of the sequencing and extraction, software used, and materials provided to allow replication by others?

Yes

Are the rationale for sequencing the genome and the species significance clearly described?

Yes

Are the protocols appropriate and is the work technically sound?

Yes

Reviewer Expertise:

Genomics and transcriptomics

I confirm that I have read this submission and believe that I have an appropriate level of expertise to confirm that it is of an acceptable scientific standard.

# Reviewer report: Reviewer response for version 1

**Published:** 2026-09-18

**Authors:** Automated Benchmarking

**Affiliations:** 1 Wellcome Sanger Institute, Hinxton, UK

As this article is a Genome Note, Automated Benchmarking has been used to determine whether the reported assembly passes four defined checks established by the Earth BioGenome Project: the raw and assembled data are publicly available, the Earth BioGenome Project three-part metric is met, the k-mer completeness exceeds 90%, and the BUSCO score passes the stated threshold. For Epilobium ciliatum (drEpiCili1), all four checks passed. Data available: pass. ENA study PRJEB81636, raw-read accessions ERR13900460, ERR13907240, and ERR14379151, and assembly accessions GCA_965637465.1 and GCA_965637425.1 are public. Earth BioGenome Project three-part metric: pass. Haplotype 1 is reported as 6.C.Q65, meeting the 6.C.Q40 reference standard. k-mer completeness: pass. The combined haplotype assemblies have 97.8% k-mer completeness, exceeding the Earth BioGenome Project target of 90%. BUSCO: pass. Haplotype 1, which is proposed as the reference assembly for this species, has 96.5% complete BUSCOs (84.2% single-copy and 12.3% duplicated). BUSCO software version: 6.0.0; lineage dataset: eudicots_odb10. It meets the plant-specific target of greater than 90% complete BUSCOs. This decision uses values reported in the Genome Note and public accession records.

Reviewer Expertise:

NA

I confirm that I have read this submission and believe that I have an appropriate level of expertise to confirm that it is of an acceptable scientific standard.
